# Systematic Study of the Physicochemical Properties of a Homologous Series of Aminobisphosphonates

**DOI:** 10.3390/molecules170910928

**Published:** 2012-09-12

**Authors:** Aino-Liisa Alanne, Helena Hyvönen, Manu Lahtinen, Markku Ylisirniö, Petri Turhanen, Erkki Kolehmainen, Sirpa Peräniemi, Jouko Vepsäläinen

**Affiliations:** 1 School of Pharmacy, University of Eastern Finland, Biocenter Kuopio, P.O. Box 1627, FIN-70211 Kuopio, Finland; 2 Laboratory of Inorganic Chemistry, Department of Chemistry, University of Helsinki, P.O. Box 55, FIN-00014 Helsinki, Finland; 3 Department of Chemistry, University of Jyväskylä, P.O. Box 35, FIN-40014 Jyväskylä, Finland

**Keywords:** bisphosphonates, aqueous solubility, pKa, physicochemical properties

## Abstract

Aminobisphosphonates, e.g., alendronate and neridronate, are a well known class of molecules used as drugs for various bone diseases. Although these molecules have been available for decades, a detailed understanding of their most important physicochemical properties under comparable conditions is lacking. In this study, ten aminobisphosphonates, H_2_N(CH_2_)_n_C(OH)[P(O)(OH)_2_]_2_, in which n = 2–5, 7–11 and 15 have been synthesized. Their aqueous solubility as a function of temperature and pH, pK_a_-values, thermal stability, IR absorptions, and NMR spectral data for both liquid (^1^H, ^13^C, ^31^P-NMR) and solid state (^13^C, ^15^N and ^31^P-CPMAS NMR) were determined.

## 1. Introduction

Bisphosphonates (BPs), characterized by the presence of the P-C-P backbone, are stable analogues of naturally occurring pyrophosphate. The BPs were first synthesized about 150 years ago [1], but the golden age of these molecules started approximately 50 years ago [2]. Initially BPs were used as water softeners and in prevention of scaling, but their current therapeutic use stems from their high affinity for the bone mineral hydroxyapatite. In the clinic, BPs are mainly used for treatment of different bone diseases and calcium metabolism disorders [3,4], but they are also effective as bone imaging agents when linked to a gamma-emitting technetium isotope. They can also be bone-targeting promoieties, e.g., for anti-inflammatory drugs [5], as solvent extraction reagents for actinide ions [6] and are even present in a new class of herbicides [7]. Recently, BPs have been used as growth inhibitors for parasitic diseases like malaria [8] and in crystal engineering studies [9]. BPs have been also reported to be effective inhibitors of atherosclerosis [10] and nitrogen-containing BPs have been shown to be effective antitumor agents [11].

The medical use BPs can be divided into two categories, non-nitrogen-containing (NNBP) and nitrogen-containing (NBP) molecules, based on their different mechanism of action in living systems [12]. The NNBP compounds (e.g., etidronate, clodronate) lack a nitrogen atom at the bridging carbon substituents, whereas NBP compounds (e.g., pamidronate, alendronate and zoledronate) have at least one nitrogen atom incorporated at the bridging carbon side-chains. Moreover, BPs are also classified based on their potency in different generations. Typical examples of 1st generation BPs are the NNBP compounds mentioned above. NBP compounds with sp^3^ nitrogen as a part of alkyl side-chain are called 2nd generation compounds while the 3rd generation NBP compounds are those molecules in which the sp^2^ nitrogen is a part of a heterocyclic ring. The studied compounds here belong to the 2nd generation BPs.

Our group has studied BP compounds for more than 20 years. Recently, we became interested in preparing a series of aminobisphosphonates (ABPs) with variable chain lengths (n = 2–15), since it was obvious that the chain length could influence the solubility, *i.e.*, pamidronate (**1**) was almost freely soluble in water whereas a compound with a longer chain, e.g., n = 10, was virtually insoluble in water and most common organic solvents. Moreover, although pamidronate (**1**), alendronate (**2**) and neridronate (**4**) are in drug use and they have been intensively studied by several groups, there is still some discrepant information, concerning their properties, e.g., there are variations in their pK_a_ values published in the literature [13,14,15,16,17,18,19].

**Figure 1:**
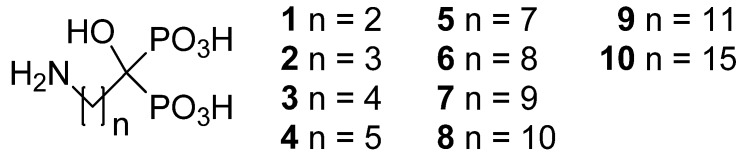
Structures of studied aminobisphosphonates.

In this work, we describe the syntheses of the ten ABPs shown in Figure 1. Their aqueous solubilities and pK_a_-values as a function of carbon chain length were determined. The compounds were also characterized by IR and NMR spectroscopic methods. NMR spectra were recorded both for liquid (^1^H, ^13^C, ^31^P-NMR) and solid state samples (^13^C, ^15^N and ^31^P-NMR). Elemental analyses were measured determine out the number of water molecules (hydrates) in the samples and this data was confirmed with thermal analysis. We also attempted to measure accurate logP values (−1 to −4) with the conventional water-octanol system, but the solubility of ABPs in the octanol phase was minimal and it was thus difficult to obtain accurate reproducible values.

## 2. Results and Discussion

### 2.1. Solubility of the ABPs

The studied compounds **1**–**10** were prepared according to the general method reported by Kieczykowski *et al.* [20]. All the compounds were readily obtained with >98% purity after crystallization from ethanol, water or a mixture of ethanol and water. Yields were typically between 75–100%, except for pamidronic acid (**1**) which was crystallized as a monosodium salt in 22% yield. Preparation of the compound with n = 6 was not successful using this method and the starting materials needed for compounds in which n = 12–14 were rather expensive and therefore not used.

Determination of the exact molecular composition (formula) was the key to determining the physicochemical data for the studied compounds exactly, since based on the earlier studies, e.g., for different clodronic acid derivatives [21] it is known that BPs may crystallize as hydrates containing up to five molecules of water. The number of water molecules in the studied compounds were measured using the traditional elemental analysis technique and the results were verified by thermal decomposition analysis. Table 1 provides the formulas of the amino(CH_2_)_n_bisphosphonates, their pH’s at saturated solution, and aqueous solubilities at 21 °C. However, only compounds **6** and **8** contained one water molecule each, even though the compounds were stored for days in an open vial.

**Table 1:** Prepared aminobisphosphonates with the formula determined from elemental analysis, the pH of their saturated solutions, and the aqueous solubilities at 21°C (agitation time 30 min).

Compound	n	Formula	MW	pH	Solubility	
					(mg P/L) ^a^	(mgL) ^b^
1	2	C_3_H_10_NO_7_P_2_Na	257.05	4.62	2634	10928
2	3	C_4_H_13_NO_7_P_2_	249.10	1.94	1905	7663
3	4	C_5_H_15_NO_7_P_2_	263.12	2.22	944	4009
4	5	C_6_H_17_NO_7_P_2_	277.15	2.26	764	3419
5	7	C_8_H_21_NO_7_P_2_	305.20	2.61	426	2099
6	8	C_9_H_23_NO_7_P_2_ · H_2_O	337.23	2.51	400	2066
7	9	C_10_H_25_NO_7_P_2_	333.26	3.28	40	217
8	10	C_11_H_27_NO_7_P_2_ · H_2_O	365.30	4.71	10	58
9	11	C_12_H_29_NO_7_P_2_	361.31	4.05	5	30
10	15	C_16_H_37_NO_7_P_2_	417.42	7.79	6	39

^a^ phosphorus concentration in saturated solution; ^b^ compound solubility.

The accurate determination of solubility for the compounds here is demanding since the solubility is dependent on several factors, such as crystal size, stirring efficiency and stirring time. The values stated in Table 1 are good estimates for solubility, since the values in the table were measured under comparable conditions (temperature, stirring speed and time) for all the compounds, which were prepared and crystallized using the same protocol. However, compound **1** was crystallized as a monosodium salt which presumably made it more soluble than it would have been in the acidic form. The solubility for the studied compounds here are somewhat less than the solubility measured as a function of temperature using different agitation times of 30 min and 24 h, respectively. In general, in terms of water solubility, the studied compounds can be classified into three groups depending on the chain length: compounds **1**–**6** are soluble in gram quantities, compound **7** in quantities of hundreds of milligrams and the remaining compounds in tens of milligrams per liter (Figure S1). Most probably the reason for the poor solubility of the compounds with the longer carbon chains (n > 7) is increased hydrophobicity and carbon chain van der Waals forces that overcome hydrogen bonding forces between water and the heteroatoms in the ABPs.

**Figure 2:**
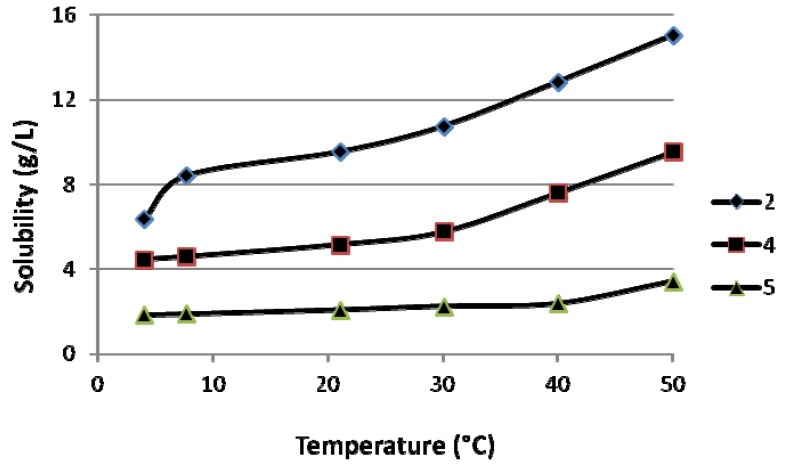
The effect of temperature on the aqueous solubility (mg/L) of ABPs **2**, **4** and **5** (agitation time 30 min).

The effect of temperature on solubility was also studied for compounds **2**, **4** and **5** at 4.0 °C, 7.6 °C, 21.0 °C, 30.0 °C, 40.0 °C and 50.0 °C (Table S1). As expected, the solubility increased when the temperature was increased as shown in Figure 2. Interestingly, solubility was increased markedly for compounds **2** and **4** after 30 °C but for compound **5** the same effect was observed after 40.0 °C. In general, the solubility for the compounds was almost doubled, when the temperature was increased to about 40.0 °C, in the temperature transition from 7.6 °C to 50.0 °C. 

**Figure 3:**
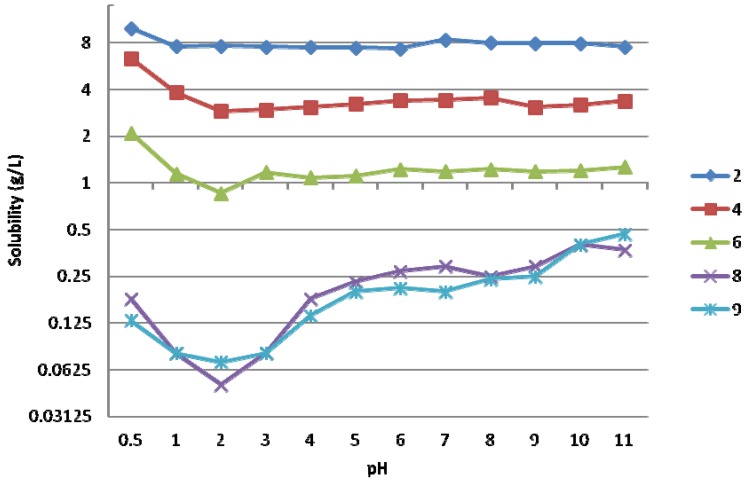
The effect of pH on the aqueous solubility (mg/L) of BP (a) **2**, **4** and **6**; (b) **8** and **9** (agitation time 30 min).

As is shown in Figure 3, the effect of pH from 0.5 to 11.0 on solubility was studied for compounds **2**, **4**, **6**, **8** and **9** (Table S2). Interestingly, pH had no major influence on the solubility of compounds **2**, **4** and **6** and only at very low pH (pH 0.5), were the solubilities doubled as compared to the values at pH 1. Instead, compounds **8** and **9** which had low solubilities displayed a clear solubility minimum at about pH 2 with the values increasing at both low and elevated pH’s.

### 2.2. Protonation Constants for Compounds *1–5*

The analysis of the data was initiated by plotting Z_H_*versus* pH [see Equation (3) and Figure S2 in the Supporting Information]. The neutralization titrations show that the stepwise deprotonation of H_5_L^+^ to H_3_L^−^ occurred in a very acidic pH range from values less than 1 to about 3 (Z_H_ from 3 to 1). H_3_L^−^ is the major species from pH 3 to pH 5 when Z_H_ = 1. H_2_L^2−^ is the major species from pH 7 to pH 10 when Z_H_ = 0 (three phosphonic acid groups). The negative Z_H_ values that were obtained in the pH range from 10 to over 12 reveal that in alkaline solution, one proton can leave the ligands from the last phosphonic acid group, H_2_L^2−^ to HL^3−^, and finally from the amino group, HL^3−^ to L^4−^. In Figure S2 in the Supporting Information, the symbols show the measured values and solid lines are the Z_H_ curves calculated from log β values. 

Results were obtained from SUPERQUAD as overall stability constants (log β values); following the chosen zero level H_2_L^2−^. This zero level H_2_L^2−^ was chosen in the calculations to avoid any accumulation of errors of protonation constants at different pH ranges. The log β results for reactions (4) and (5) obtained in the final refinements are listed in Table S3 (see Supporting Information) with error limits (±3σ), number of points/number of titrations and χ^2^/Σ statistics. All results for compounds **1**, **2**, **3**, **4**, and **5** were recalculated for the stepwise protonation constants, log K (or pKa) values, see Table 2. Although the values of the last protonation constants (pKa_1_ values) could be calculated from the experimental data, they should only be considered as approximate values. This is reflected also in the larger error limits for these constants as compared to the other constants. 

The logK values for compounds **1**, **2** and **4** from earlier studies can be found in literature, but they are not entirely comparable with each other because of the different experimental conditions used (e.g., background electrolyte, ionic strength, temperature or method) [13,14,15,16,17,18,19]. Although these values vary to some extent for each ligand, they do agree with each other on the whole, taking into consideration the different conditions. Furthermore, the results of this study correspond well to the literature values for compounds **1**, **2** and **4**. A detailed comparison of literature pK_a_ values is given in Table 3 and Table 4.

**Table 2:** The stepwise protonation of compounds **1**, **2**, **3**, **4**, and **5** in this study.

Protonation Reaction	log K					pKa
	1	2	3	4	5	
L^4−^ + H^+^⇋ HL^3−^	12.86 ^a^	12.13 ^f^	12.05	11.94 ^k^	11.65	pKa_5_
HL^3−^ + H^+^⇋ H_2_L^2−^	10.04 ^b^	10.69 ^g^	10.78	10.86 ^l^	10.67	pKa_4_
H_2_L^2−^ + H^+^⇋ H_3_L^−^	5.90 ^c^	6.26 ^h^	6.44	6.62 ^m^	6.75	pKa_3_
H_3_L^−^ + H^+^⇋ H_4_L	1.70 ^d^	2.12 ^i^	2.30	2.38 ^n^	2.52	pKa_2_
H_4_L + H^+^⇋ H_5_L^+^	1.06 ^e^	0.60 ^j^	0.62	0.94	1.08	pKa_1_

Variations in the pK_a_ values in the literature for compounds 1, 2 and 4: ^a^ 13.06–10.40; ^b^ 10.30–9.46; ^c^ 6.04–5.39; ^d^ 2.56–1.80; ^e^ 1.24 or lower; ^f^ 12.68–10.5; ^g^ 11.07–10.25; ^h^ 8.73–5.95; ^i^ 2.72–2.16; ^j^ 1.33 or lower; ^k^ 10.9; ^l^ 10.66–8.63; ^m^ 6.50–5.49; ^n^ 2.90–2.45.

The following general trend can be found in the results of this study: the lengthening of the CH_2_ chain between BP and amino groups decreases the value of the first protonation constant (pKa_5_, amino group) and increases the values of the other protonation constants (phosphonate groups) in most cases (Table 2). The same trend can be seen also in those earlier studies where a comparison is possible, e.g., for all pK_a_ values with compounds **1** and **2** [14,17] and for pKa values of the phosphonate groups with compounds **1** and **2** [13,18] and with compounds **1** and **4** [16]. The protonation of the ligands is also illustrated in the percentage distribution curves calculated from log β values (see Figure S3 in the Supporting Information).

**Table 3:** The stepwise protonation of compound **1** in the literature [4,13,14,15,16,17,18].

Protonation Reaction	compound 1 logK							pKa
	Ref.13	Ref.14	Ref.15	Ref.4	Ref.17	Ref.18	Ref.18	
L^4−^ + H^+^⇋ HL^3−^	10.8	13.06	10.95	11.02	12.14	10.74	10.40	pKa_5_
HL^3–^ + H^+^⇋ H_2_L^2−^	9.9	10.30	9.80	9.90	10.18	9.97	9.46	pKa_4_
H_2_L^2−^ + H^+^⇋ H_3_L^−^	5.83	5.85	6.01	5.86	6.04	6.01	5.39	pKa_3_
H_3_L^−^ + H^+^⇋ H_4_L	2.55	1.80	2.56	2.04	1.93	-	-	pKa_2_
H_4_L + H^+^⇋ H_5_L^+^	-	<1.2	-	-	1.24	-	-	pKa_1_

**Table 4:** The stepwise protonation of compounds **2** and **4** in the literature [4,13,14,17,18,19].

Protonation Reaction	Comp. 2 logK						Comp. 4 logK		pKa
	Ref.13	Ref.14	Ref.17	Ref.18	Ref.18	Ref.19	Ref.13	Ref.4	
L^4−^ + H^+^⇋ HL^3−^	11.6	12.68	11.82	11.4	10.5	12.04	10.9	-	pKa_5_
HL^3−^ + H^+^⇋ H_2_L^2−^	10.5	11.07	10.96	10.68	10.25	10.77	8.63	10.66	pKa_4_
H_2_L^2−^ + H^+^⇋ H_3_L^−^	8.73	6.36	6.39	6.38	5.95	6.21	5.49	6.50	pKa_3_
H_3_L^−^ + H^+^⇋ H_4_L	2.72	2.19	2.22	2.24	2.34	2.16	2.90	2.45	pKa_2_
H_4_L + H^+^⇋ H_5_L^+^	-	<1.2	1.33	-	-	~1	-	-	pKa_1_

The pK_a_ values were also determined for compounds **1**–**5** using the Sirius instrument. This comparison was done since this instrument is commonly used for the determination of pK_a_ values in many laboratories and we wanted to study the accuracy and reliability of this automatic system. In general, in our hands the Sirius instrument was not able to measure the first pK_a1_ values near to 1, but rather reliable values for pK_a2_ and pK_a3_ values were observed for all studied compounds. In addition, for compounds **1** and **2**, the remaining pK_a_ values were consistent with the values shown in Table 2, but for compounds **3**–**5** clear differences were observed. Detailed results are shown in the Supporting Information (Table S4).

### 2.3. NMR Spectroscopy

^1^H, ^13^C and ^31^P-NMR spectroscopy are the best methods with which to analyze the chemical composition of BPs in liquid and solid states. However, liquid state NMR is more common and solid state measurements are typically used for poorly soluble compounds. The ^13^C and ^31^P-NMR chemical shift data both in liquid and solid states including also the solid state ^15^N-NMR chemical shifts for the studied compounds are given in Table S5 in the Supporting Information. Detailed ^1^H-NMR data is also given in the Supporting Information.

The ^13^C-NMR signals for the studied ABPs were easily assigned when starting from the bridging carbon resonance, which appeared as a characteristic triplet at approx. 79 ppm and 72–75 ppm in liquid and solid state spectra, respectively, due to the presence of deshielding OH group and the coupling to two phosphorus atoms. C-3 also appeared as a triplet (^3^*J*_CP_ ca. 5 Hz) in the liquid state and C-2 was clearly broadened (^2^*J*_CP_ < 1 Hz) as compared to other carbon signals due to phosphorous coupling. The assignment of carbon next to the amino group (CH_2_-N) was also straight forward and its shift varied from 41 to 45 and 38 to 42 ppm in liquid and solid state spectra, respectively. The rest of the chemical shifts were as expected, but it was impossible to assign the chemical shifts for compounds **6**–**10** in the solid state due to overlapping shifts and polymorphism which broadened the observed signals.

^15^N-CPMAS chemical shifts are typical for primary amines and do not vary extensively (Table S5). All liquid state ^31^P-NMR signals are also approx. at 19 ppm, this being a typical value for BP compounds. However, in the solid state ^31^P-NMR spectra, two signals for each compound were observed, since in the crystalline state the phosphorous atoms are always located in an unequal chemical environment. The ^31^P-NMR chemical shifts for acids **2**–**4** (compound **1** is monosodium salt) were also clearly different compared to acids **5**–**10** indicating that these groups have different crystal structures from each other. 

**Figure 4:**
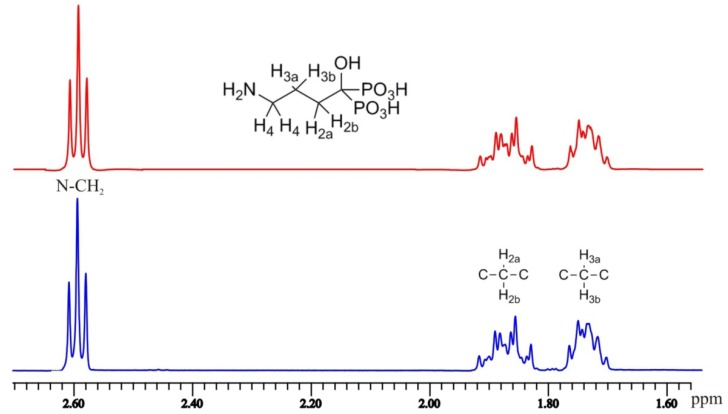
Observed (bottom) and calculated (upper) ^1^H-NMR spectrum of alendronic acid.

A detailed analysis of ^1^H-NMR spectra for the studied compounds without iterative computerized methods is impossible, since the spin systems in compounds like these are more complicated than expected because of prochirality. It is commonly claimed in the literature that, for certain BPs, e.g., alendronate, the N-CH_2_-CH_2_-CH_2_-C(OH)P_2_ gives rise to a first order spin system, in which NCH_2_ group exists as a triplet (t), the middle CH_2_ as a quintet and the CH_2_-C group as t+t (last triplet due to phosphorous coupling). However, as shown in Figure 4, only the NCH_2_ group appears as a first order triplet with expected 1:2:1 intensities and the rest of CH_2_ groups give rise to a complicated multiplet. In fact, the R-CH_2_-C(OH)P_2_ fragment is a typical example of prochiral systems, in which CH_2_ protons become magnetically non-equivalent, forming an AB-spin system and they likely have different chemical shifts and coupling constants [22]. The same is true at least for the protons at the next CH_2_ group.

The results from computerized analysis are given in Table 5 for compounds **1**–**5**. A detailed analysis for the rest of the compounds was not realistic due to the broad signals and heavily overlapping peaks. Moreover, the values given in Table 5 are only good estimates for the coupling constants present in the studied molecules, since the spectral lines were rather broad (W_1/2_ = 1–1.5 Hz) due intra- and intermolecular hydrogen bonding. Iterative analysis showed clearly that CH_2_ protons had the same chemical shift, since geminal ^2^*J*_HH_ coupling was missing, but different vicinal ^3^*J*_HH_ coupling constants existed. ^3^*J*_HP_ coupling constants were taken from coupled ^31^P-NMR spectra and ^4^*J*_HP_ coupling constants were obtained from spectral analysis.

**Table 5:** ^1^H-NMR chemical shifts and coupling constant for compounds **1**–**5**.

		H-2a	H-2b	H-3a	H-3b	H-4	H-5	H-6
**1**	shift (ppm)^3^*J*_HH_ (Hz)^3/4^*J*_HP_ (Hz)	2.04 9.8, 5.8 13.4	2.04 10.0, 5.7 13.4	2.94 10.0, 5.8 0.9	2.94 9.8, 5.7 0.9			
**2**	shift (ppm)^3^*J*_HH_ (Hz)^3/4^*J*_HP_ (Hz)	1.87 13.9, 4.4 13.5	1.87 11.7, 4.2 13.5	1.73 11.7, 8.8, 4.4 1.0	1.73 13.9, 5.4, 4.2 1.0	2.59 8.8, 5.4-		
**3**	shift (ppm)^3^*J*_HH_ (Hz)^3/4^*J*_HP_ (Hz)	1.88 13.9, 5.6 ^1^13.6	1.88 11.8, 2.9 13.6	1,62 13.9, 5.9, 2.9 1.0	1.62 11.8, 9.4, 5.6 1.0	1.47 9.4, 6.9, 5.9 -	2.70 6.9-	
**4**	shift (ppm)^3^*J*_HH_ (Hz)^3/4^*J*_HP_ (Hz)	1.88 12.4, 4.0 13.3	1.88 13.4, 4.5 13.3	1.58 13.4, 7.2, 4.0 0.9	1.58 12.4, 7.9, 4.5 0.9	1.29 7.9, 7.8, 7.2-	1.47 7.8, 7.1-	2.61 7.1-
**5**	shift (ppm)^3^*J*_HH_ (Hz)^3/4^*J*_HP_ (Hz)	1.87 13.0, 4.313.3	1.87 13.0, 4.3 13.6	1.56 13.0, 6.0, 4.3 1.5	1.56 13.0, 7.7, 4.3 1.5	^2^	^2^	1.46 7.1, 6.7-

^1^^4^J_2a4_ = 1.9 Hz ^2^ H-4 and H-5 signals not analyzed due to the presence of heavily overlapping signals, H-7: 2.62 ppm, ^3^*J*_HH_ = 7.1 Hz.

The results clearly indicate that the R and C(OH)P_2_ groups are almost entirely *trans* to each other in the R-CH_2_-CH_2_-C(OH)P_2_ part of the molecule containing three or more CH_2_ units between the amino and C(OH)P_2_ moieties, since both CH_2_ protons have one large *trans* (>11 Hz) and one *gauche* (4–6 Hz) coupling. In the case of pamidronate, coupling constants are about 6 and 10 Hz indicating that the amount of the trans conformation is diminished due to intramolecular hydrogen bonding and zwitter ion interaction between amino and phosphorous groups.

### 2.4. Thermal Analysis

Despite the fact that alkylaminobis(phosphonates) are a group of substances that have been widely investigated and are medically used substances, according to the literature, their thermoanalytical properties have been rarely determined in a comprehensive manner. In this study, thermoanalytical methods were utilized, in order to evaluate the thermal stability and potential hydrate stoichiometry of the examined compounds. Moreover, thermogravimetric studies were performed in parallel using two instruments with slightly different sample crucibles (tall and a shallow vessel), in order to evaluate the influence of “micro-atmospheric effects” that may prevail during heating inside the different crucibles used in the measurements.

For compounds which have a primary amine and an acidic phosphonate group in the same molecule, this enables the formation of zwitterions, at least for compounds having shorter alkylidene chains between the terminal groups. Indeed a few X-ray crystallographic studies have been conducted to reveal the inner salt nature of pamidronic (1-hydroxypropylidene chain) alendronic (1-hydroxybutylidene) and neridronic (1-hydroxyhexylidene) acids [23,24,25]. A similar zwitterionic behavior is generally observed also in aliphatic amino acids, the decomposition of which is typically initiated either via deamination or decarboxylation reaction, of which the latter is the more favored route among this type of amino acids [26]. With alkylaminobis(phosphonates), it is anticipated that the decomposition may more likely commence via deamination instead of a dephosphonation reaction due to the more stable nature of the phosphonate group. One also has to take into account the fact that special care must be taken in evaluating the potentiality of water molecules in these types of compounds, because the weight loss difference between cleavage of ammonia and one water crystal is nearly always the same for substances having formula weights around 300 g/mol as in this case. Therefore, a truly anhydrous substance can easily be misinterpreted as being a hydrate and *vice versa* when the analysis of the evolved gas components is not available. Therefore, it is crucial to apply in parallel to TGA, other characterization techniques as well. In this study, elemental analysis (see preceding sections), visual inspection in the melting apparatus and structural studies by X-ray powder diffraction were applied. It is noted that the successful powder structure determination of compounds **3** and **5** will be published elsewhere as they were not within the scope of this study.

The experimental and calculated Δwt-%, together with the temperature range of dehydration, decomposition onsets and estimated melting points (from DTA signal) can all be seen in Table 6 and the measured TG curves are given in the Supporting Information. The ABPs **1**–**5** with shorter 1-hydroxyalkylidene chains (varying from 2 to 7 carbons) all exhibited a weight loss of about 4–7 wt-% on a first step with temperature onsets varying from about 150 to 230 °C. It might seem that weight losses are in good agreement with the calculated cleavage values of one water of crystallization molecule present in each compound. However, as evidenced by visual monitoring in the melting point apparatus, elemental analyses, and by X-ray powder diffraction analyses all five compounds proved to be free of water of crystallization. Consequently, the first decomposition process for these compounds is in good agreement with the proposal that it is due to the cleavage of an amino group as NH_3_ (e.g., for compound **2** (exp. 7.02% calc. 6.84%; see detailed values for each compound in the Supporting information Table S6). The second decomposition step of **2**–**5** readily followed the first step and this is assumed to be due to the release of water (e.g., for **2** exp. 7.49% calc. 7.23%) which most likely originates from the cleavage of the hydroxyl group attached to C1-carbon. For **2**–**5**, the major degradation reactions commence in a compound-specific manner above 250–300 °C with breakage of the C-P bond that leads to further pyrolysis of the samples [27]. It can be noted that overall the shorter alkyl chain compounds display somewhat lower decomposition temperatures than the longer alkyl chain versions (Table 6). In case of compound **1** (monosodium salt), the decomposition deviates slightly from the acids **2**–**5**, as after the cleavage of the amino group between 100–200 °C, the subsequent larger weight loss (~14.2 wt-%) includes most likely the cleavage of the hydroxyl group as in **2**–**5** but concurrently, a more comprehensive degradation of the compound occurs in the temperature range of 200–300 °C. This large weight loss step is followed by a further pyrolysis of the product at a temperature above 300 °C. In addition, the higher residual weight at 700 °C is indicative of the formation of inorganic thermally stable Na-containing salt(s) together with charring. In an attempt to identify the black residue, X-ray powder diffraction data was evaluated, and as a result, sharp diffraction peaks characteristic for disodium diphosphate (Na_2_H_2_P_2_O_7_) were identified in the pattern along with broad scattering humps caused by the amorphous carbonized content (Supporting Information, Figure S4).

**Table 6:** Thermal properties of compounds **1**−**10**.

Comp.	MW	Δwt-% (%)		Temp. range ^b^	*T_m _* ^c^	*T_d _* ^d^	Ref. melting points
		exp.	calc.				
1	257.05	anhydrous			149	152	-
2	249.10	anhydrous			223	224	234 (dec.) ^f^
3	263.12	anhydrous			216	220	212 (dec.) ^f^
4	277.15	anhydrous			205	212	247^ f^
5	305.20	anhydrous			193	195	-
6	337.23 ^a^	5.00	5.34 (1 H_2_O)	33–175 (81)	134	191	-
7	333.26	anhydrous			181	187	-
8	365.30 ^a^	4.43	4.93 (1 H_2_O)	40–148 (72)	127	202	-
9	361.31	anhydrous			181	188	-
10	417.42	anhydrous			175	180	-

^a^ MW of hydrate; ^b^ temperature range of dehydration (onset *T* in parentheses); ^c^ from DTA signal of TG/DTA (decomposition coexists with melting on all compounds); ^d^ onset values; All temperatures are in °C and the *T_d_* results are shown as an average from both instruments. Values from the individual devices can be found in Supporting Information (Table S6); ^f^ see reference [28].

For compounds **6**–**10** with long alkyl chains, visual monitoring and the elemental analysis results indicate that only **6** and **8** are monohydrates (exp. 5.00 and 4.33 wt-% calc. 5.34 and 4.93 wt-%, respectively), whereas the others exist in an anhydrous form. For compounds **7**, **9** and **10** the first weight loss step observed in the thermograms points to cleavage of the amino group, which occurs typically at about 200 °C, whereas the dehydration of compounds **6** and **8** occurs at significantly lower temperatures. In compound **6**, dehydration is initiated at ~90 °C and this is readily followed by the cleavage of the amino group. Compound **8** exhibits a similar dehydration sequence with the exception that the deamination is initiated at the higher temperature of 200 °C (Table 6). The major decomposition stage (after cleavage of amino group) of these longer alkyl chained bisphosphonates occurs quite uniformly above 350 °C.

The influence of sample crucible was clearly evidenced in the TG analyses performed as somewhat different TG curve trends were observed at higher temperature ranges for all samples. As expected, the higher crucible used in TG/DTA seems to generate a less oxidizing environment above the sample surface. This is clearly visible in the measured TG curves as the residual weights (Figure S5) at the end the temperatures were always higher in the TG/DTA in contrast to the measurements made with TGA 7, and a shallower sample crucible (Table 6). Therefore, this suggests that there is a higher degree of charring of the samples in the case of the TG/DTA instrument. This was also noted visually. Although conditions seemed to be less oxidizing in the higher crucible, all samples degraded at a higher rate within the temperature range of ~350 to 550 °C, in contrast to the situation if the samples were heated in the shallow crucible (Figure S5). It can be assumed that somewhat better oxidation conditions were present in the shallow crucible, which may have caused the formation of temporarily-stable intermediate products, which then decomposed rapidly above 550 °C, thus surpassing the values of the weight residues (Table S6) found in TG/DTA run already at ~600 °C and terminating at near to zero weight at 700 °C. It can also be postulated that the evaporation of potential oxidation products of phosphorus at higher temperatures occurred more easily in a shallow crucible, in turn speeding up the weight loss. Finally, the TG curves were essentially identical in both instruments over a temperature range varying from 100 to 250 °C, indicating that dehydration/deamination processes along with the first steps of degradation were not affected by any micro-atmospheric effects.

Finally, melting temperatures of the compounds were evaluated from the DTA signals and were compared to the literature values, when available. With the anhydrous compounds, a decreasing trend in melting temperatures was observed in conjunction with an increase in the alkylidene chain lengths, as the highest melting point (223 °C) was determined for **2** and the lowest (175 °C) for **10**. In the case of the two monohydrates, **6** and **8**, the melting occurred concurrently with dehydration process that was observed at about 130 °C for both compounds. However, it should also be noted that nearly all melting endotherms seen on DTA curves overlapped significantly with coexisting dehydration/deamination and/or decomposition transitions, inevitably conferring some degree of inaccuracy in the determination of the melting temperatures.

## 3. Experimental

### 3.1. Materials and Instrumentation

4-Aminobutyric acid (97%), 8-aminocaprylic acid (99%), 12-aminododecanoic acid (95%), 5-aminovaleric acid (97%), β-alanine (98%), 9-bromo-1-nonanol (95%), 10-bromodecanoic acid (95%), 16-bromohexadecanoic acid (>99%), phosphorus acid (99%), methanesulfonic acid (>99.5%) and phosphorus trichloride (99%) were purchased from Sigma-Aldrich (Steinheim, Germany). 6-Amino-caproic acid (>99%) and 11-aminoundecanoic acid (97%) were purchased from Acros Organics (Geel, Belgium). D_2_O (99.90% D) used in the NMR measurements was purchased from Euriso-Top (Gif-Sur-Yvette, France). Aqueous 0.1 M NaOH was prepared from Titrisol ampoules (Merck, Darmstadt, Germany) and 0.1 M HNO_3_ from Convol ampoules (BDH, Mumbai, India). The background solution was prepared by dissolving NaNO_3_ in deionised water. NaNO_3_ and NaCl used in reference electrode were p.a. grade (Merck). The water used in the dilutions and titration solutions was purified with Milli-RO and Milli-Q water purification systems (Millipore, Billerica, USA).

Infrared spectra were recorded on a Thermo Nicolet Nexus 470 FTIR spectrometer (Thermo Electron Corporation, Vantaa, Finland) with the KBr pellet technique. Liquid state ^1^H, ^13^C and ^31^P-NMR spectra were recorded on a Bruker Avance DRX spectrometer (Bruker, Karlsruhe, Germany) operating at 500.1, 125.8 and 202.5 MHz, respectively. Measurements were performed at 300 K by using standard pulse sequences and sodium 3-(trimethylsilyl)-1-propionic acid (TSP) was used as the internal standard in D_2_O. The solid state ^13^C, ^15^N and ^31^P-CPMAS NMR spectra were recorded at 300 K with a Bruker Avance 400 spectrometer operating at 100.62, 44.55 and 161.98 MHz, respectively. The spectrometer was equipped with a dual CPMAS probehead and samples were packed in 4.0 mm zirconia rotors. The contact times for the CPMAS experiments were 2 ms for ^13^C, 3 ms for ^15^N and 5 ms for ^31^P. The relaxation delay was 5 s and the spin rate was 10 KHz. In the high-power broad band, ^1^H decoupling the pulse program was spinal64 for ^13^C and ^15^N, and tppm15 for ^31^P. Typically one hundred scans were completed for ^13^C and ^31^P, and thousands for ^15^N. The ^13^C and ^15^N chemical shifts were referenced to those of glycine (176.03 ppm for carbonyl carbon and −347.3 ppm for NH_2_) measured prior to each sample. The ^31^P-NMR chemical shifts are referenced to that of sodium alendronate measured before. All computerized spectral analysis was performed using PERCH NMR software, version 2010.1 (PERCH Solution Ltd, Kuopio, Finland). The starting materials for **6**, **7** and **10** were synthesized according to Amara *et al.* [29].

### 3.2. Synthesis

Compounds were prepared according to the general method reported by Kieczykowski *et al.* [20], exemplified by the preparation of *(4-amino-1-hydroxybutylidene)bisphosphonic acid* (**2**; CAS Registry Number 66376-36-1): 4-aminobutyric acid (2.5 g, 24.2 mmol), phosphorus acid (2.0 g, 24.2 mmol) and methanesulfonic acid (11 mL) were put in a flask with a reflux condenser and a CaCl_2_-tube and the mixture was heated to 65 °C. PCl_3_ (4.3 mL, 48.4 mmol) was added dropwise and the mixture was maintained at 65–70 °C for 18 h (generally 16–20 h). Water (25 mL) was added to the cooled mixture and the solution was refluxed for 5 h. Some of the water was evaporated, EtOH was added and the product was allowed to crystallize at 8 °C. The product was collected by filtration and washed with EtOH yielding 5.1 g of **2** (85%) as white solid, yield 85%; ^1^H-NMR (D_2_O/NaOD, 500.1 MHz): δ 2.59 (2H, t, *J* = 7.0 Hz), 1.93–1.82 (2H, m), 1.78–1.69 (2H, m); ^13^C-NMR (D_2_O/NaOD, 125.8 MHz): 79.3 (t, ^1^*J*_CP_ = 134.5 Hz), 44.6, 36.2, 30.2 (t, ^2^*J*_CP_ = 5.0 Hz); ^31^P-NMR (D_2_O/NaOD, 202.5 MHz) δ 18.89 (s); IR (KBr) 3275, 2262, 1630, 1498, 1472, 1400, 1374, 1319, 1212, 1165, 1130, 1056, 994, 934, 825, 751, 725, 665 cm^−1^. Anal. Calcd. for C_4_H_13_NO_7_P_2_: C, 19.29; H, 5.26; N, 5.62; P, 24.9. Found: C, 19.19; H, 5.09; N, 5.51; P, 24.5.

*(3-Amino-1-hydroxypropylidene)bisphosphonic acid* (**1**; CAS Registry Number 40391-99-9). The pH was adjusted to 4.5 to obtain the monosodium salt, yield 22%; ^1^H-NMR (D_2_O/NaOD, 500.1 MHz) δ 2.97–2.90 (2H, t, *J* = 7.8 Hz), 2.10–1.99 (2H, m); ^13^C-NMR (D_2_O/NaOD, 125.8 MHz): 78.7 (t, ^1^*J*_CP_ = 133.8 Hz), 40.9, 40.0 (t, ^2^*J*_CP_ = 5.7 Hz); ^31^P-NMR (D_2_O/NaOD, 202.5 MHz): δ 18.46 (s); IR (KBr) 3354, 3188, 2792, 2364, 1719, 1615, 1528, 1409, 1343, 1327, 1282, 1173, 1045, 984, 937, 895, 855, 775, 715, 683, 614 cm^−1^. Anal. Calcd. for C_3_H_10_NO_7_P_2_Na: C, 14.02; H, 3.92; N, 5.45; P, 24.1. Found: C, 14.48; H, 4.41; N, 5.64; P, 23.3.

*(5-Amino-1-hydroxypentylidene)bisphosphonic acid* (**3**; CAS Registry Number 89732-96-7). Yield 86%; ^1^H-NMR (D_2_O/NaOD, 500.1 MHz): δ 2.70 (2H, t, *J* = 7.0 Hz), 1.94–1.82 (2H, m), 1.66–1.57 (2H, m), 1.51–1.43 (2H, m); ^13^C-NMR (D_2_O/NaOD, 125.8 MHz): 79.2 (t, ^1^*J*_CP_ = 133.5 Hz), 42.9, 38.1, 34.3, 24.1 (t, ^2^*J*_CP_ = 5.3 Hz); ^31^P-NMR (D_2_O/NaOD, 202.5 MHz): δ 18.92 (s); IR (KBr) 3209, 2959, 2331, 1617, 1503, 1468, 1452, 1219–939, 824, 663 cm^−1^. Anal. Calcd. for C_5_H_15_NO_7_P_2_: C, 22.82; H, 5.75; N, 5.32; P, 23.5. Found: C, 22.59; H, 5.82; N, 5.30; P, 23.5.

*(6-Amino-1-hydroxyhexylidene)bisphosphonic acid* (**4**; CAS Registry Number 79778-41-9). Yield 88%; ^1^H-NMR (D_2_O/NaOD, 500.1 MHz): δ 2.61 (2H, t, *J* = 7.0 Hz), 1.94–1.82 (2H, m), 1.62–1.53 (2H, m), 1.51–1.43 (2H, m), 1.34–1.26 (2H, m); ^13^C-NMR (D_2_O/NaOD, 125.8 MHz): 79.6 (t, ^1^*J*_CP_ = 134.4 Hz), 43.5, 39.0, 34.7, 30.4, 26.9 (t, ^2^*J*_CP_ = 5.2 Hz); ^31^P-NMR (D_2_O/NaOD, 202.5 MHz): δ 19.12 (s); IR (KBr) 3178, 2953, 2296, 1648, 1582, 1496, 1476, 1441, 1112, 1034, 997, 939, 905, 824, 777, 724, 657 cm^−1^. Anal. Calcd. for C_6_H_17_NO_7_P_2_: C, 26.00; H, 6.18; N, 5.05; P, 22.4. Found: C, 25.97; H, 6.17; N, 4.97; P, 22.2.

*(8-Amino-1-hydroxyoctylidene)bisphosphonic acid* (**5**; CAS Registry Number: 144050-49-7). Yield 75%; ^1^H-NMR (D_2_O/NaOD, 500.1 MHz): δ 2.62 (2H, t, *J* = 7.0 Hz), 1.93–1.81 (2H, m), 1.61–1.52 (2H, m), 1.50–1.41 (2H, m), 1.39–1.24 (6H, m); ^13^C-NMR (D_2_O/NaOD, 125.8 MHz): 78.9 (t, ^1^*J*_CP_ = 132.4 Hz), 43.2, 38.4, 33.6, 33.0, 31.5, 29.0, 27.0 (t, ^2^*J*_CP_ = 5.6 Hz); ^31^P-NMR (D_2_O/NaOD, 202.5 MHz): δ 19.05 (s); IR (KBr) 3327, 3150, 2901, 2848, 2276, 1650, 1620, 1541, 1471, 1398, 1334, 1300, 1224, 1151, 1079, 967, 842, 813, 767, 729, 647 cm^−1^. Anal. Calcd. for C_8_H_21_NO_7_P_2_: C, 31.48; H, 6.93; N, 4.59; P, 20.3. Found: C, 31.44; H, 6.98; N, 4.69; P, 20.5.

*(9-Amino-1-hydroxynonylidene)bisphosphonic acid* (**6**; CAS Registry Number: 144050-48-6). As an exception to the general method **6** was refluxed for 5 h in 2 M HCl instead of water and pH was adjusted to 7 to obtain the disodium salt. The product was recrystallized from water-ethanol, yield 63%; ^1^H-NMR (D_2_O/NaOD, 500.1 MHz): δ 2.59 (2H, t, *J* = 7.0 Hz), 1.93–1.79 (2H, m), 1.59–1.50 (2H, m), 1.50–1.38 (2H, m), 1.37–1.21 (8H, m); ^13^C-NMR (D_2_O/NaOD, 125.8 MHz): 79.6 (t, ^1^*J*_CP_ = 134.6 Hz), 43.4, 39.0, 34.7, 33.2, 31.9, 31.7, 28.9, 27.2 (t, ^2^*J*_CP_ = 5.2 Hz); ^31^P-NMR (D_2_O/NaOD, 202.5 MHz): δ 19.30 (s); IR (KBr) 3195, 2910, 2850, 1637, 1558, 1475, 1168, 1040, 906, 720, 672 cm^−1^. Anal. Calcd. for C_9_H_23_NO_7_P_2 _H_2_O: C, 32.05; H, 7.47; N, 4.15; P, 18.4. Found: C, 31.55; H, 6.88; N, 3.92; P, 18.6.

*(10-Amino-1-hydroxydecylidene)bisphosphonic acid* (**7**). As an exception to the general method **7** was refluxed for 5 h in 2 M HCl instead of water and the product was washed with water, yield 66%; ^1^H-NMR (D_2_O/NaOD, 500.1 MHz): δ 2.58 (2H, t, *J* = 7.0 Hz), 1.93–1.80 (2H, m), 1.60–1.50 (2H, m), 1.47–1.38 (2H, m), 1.37–1.23 (10H, m); ^13^C-NMR (D_2_O/NaOD, 125.8 MHz): 79.7 (t, ^1^*J*_CP_ = 134.5 Hz), 43.4, 39.1, 34.7, 33.3, 31.9, 31.9, 31.5, 28.9, 27.2 (t, ^2^*J*_CP_ = 5.1 Hz); ^31^P-NMR (D_2_O/NaOD, 202.5 MHz): δ 19.25 (s); IR (KBr) 3327, 2913, 2849, 2274, 1648, 1542, 1472, 1338, 1228, 1082, 970, 921, 810, 772, 742, 728, 647 cm^−1^. Anal. Calcd. for C_10_H_25_NO_7_P_2_: C, 36.04; H, 7.56; N, 4.20; P, 18.6. Found: C, 35.87; H, 7.65; N, 3.94; P, 18.2.

*(11-Amino-1-hydroxyundecylidene)bisphosphonic acid* (**8**; CAS Registry Number: 97815-71-9). Yield 100%; ^1^H-NMR (D_2_O/NaOD, 500.1 MHz): δ 2.58 (2H, t, *J* = 7.0), 1.92–1.80 (2H, m), 1.58–1.49 (2H, m), 1.46–1.37 (2H, m), 1.36–1.23 (12H, m); ^13^C-NMR (D_2_O/NaOD, 125.8 MHz): 79.6 (t, ^1^*J*_CP_ = 134.4 Hz), 43.4, 39.1, 34.6, 33.3, 31.9, 31.9, 31.6, 31.4, 28.9, 27.2 (t, ^2^*J*_CP_ = 5.2 Hz); ^31^P-NMR (D_2_O/NaOD, 202.5 MHz): δ 19.33 (s); IR (KBr) 3566, 3198, 2914, 2847, 2664, 2340, 1635, 1521, 1475, 1380, 1300, 1206, 1155, 1097, 1042, 989–930, 722, 668 cm^−1^. Anal. Calcd. for C_11_H_27_NO_7_P_2_ H_2_O: C, 36.17; H, 8.00; N, 3.83; P, 17.0. Found: C, 36.20; H, 8.03; N, 3.73; P, 17.2.

*(12-Amino-1-hydroxydodecylidene)bisphosphonic acid* (**9**; CAS Registry Number: 724457-78-7). The product was washed with hot water, yield 85%; ^1^H-NMR (D_2_O/NaOD, 500.1 MHz): δ 2.58 (2H, t, *J* = 7.0), 1.92–1.80 (2H, m), 1.58–1.49 (2H, m), 1.46–1.37 (2H, m), 1.36–1.23 (14H, m); ^13^C-NMR (D_2_O/NaOD, 125.8 MHz): 79.6 (t, ^1^*J*_CP_ = 134.4 Hz), 43.4, 39.1, 34.6, 33.3, 31.9 (2C), 31.6, 31.5, 31.4, 28.9, 27.2 (t, ^2^*J*_CP_ = 5.2 Hz); ^31^P-NMR (D_2_O/NaOD, 202.5 MHz): δ 19.35 (s); IR (KBr) 3326, 2916, 2849, 2360, 2342, 1647, 1542, 1472, 1333, 1227, 1067, 969, 935, 808, 773, 749, 730, 668, 648 cm^−1^. Anal. Calcd. for C_12_H_29_NO_7_P_2_: C, 39.89; H, 8.09; N, 3.88; P, 17.1. Found: C, 40.31; H, 8.18; N, 3.85; P, 17.0.

*(16-Amino-1-hydroxyhexadecylidene)bisphosphonic acid* (**10**). As an exception to the general method **10** was stirred for two days at 70 °C and refluxed over night after addition of water. The product was washed with methanol and hot water, yield 78%, IR 2918, 2850, 1647, 1542, 1471, 1333, 1230, 1055, 970, 916, 804, 777, 757, 728, 667 cm^−1^. Anal. Calcd. for C_16_H_37_NO_7_P_2_: C, 46.04; H, 8.93; N, 3.36; P, 14.8. Found: C, 47.41; H, 9.08; N, 3.46; P, 14.5.

### 3.3. Spectrophotometric Analysis

A Jasco V-530 UV/Vis spectrophotometer was used for phosphorus determination at 880 nm wavelength. Filtered liquid samples were diluted to the appropriate volume with Milli-Q water and were digested with the persulphate oxidation technique in acidic medium at elevated temperature to convert aminobisphosponates to orthophosphate. In an acidic medium, ammonium molybdate and antimony potassium tartrate react with orthophosphate to form a phosphomolybdic acid that is reduced to intensively colored molybdenum blue by ascorbic acid [30].

### 3.4. Determination of Solubility

Aqueous solubilities were determined at constant room temperature (21.0 °C) for compounds **1**–**10** (agitation time 30 min) and at constant 4.0 °C, 7.6 °C, 21.0 °C, 30.0 °C, 40.0 °C and 50.0 °C temperature for compounds **2**, **4** and **5** by preparing a saturated solution of the compound in Milli-Q water without buffering (agitation time 24 h). The mixture containing an excess of the studied compound was first agitated for predetermined time with magnetic stirrer and before taking the sample mixture was allowed to stand for 24 h without stirring. The sample (about 5 mL) was taken from liquid above the solids, pH was measured and the sample was filtered through 0.2 µm membrane filter to remove any possible insoluble particles. The phosphorus concentration in the filtered sample solution was determined by using the spectrophotometric method described above. Samples for pH dependent solubility measurements were prepared from compounds **2**, **4**, **6**, **8** and **9** as above, but now pH was adjusted to the desired value by using sodium hydroxide or hydrochloric acid solutions of the appropriate concentrations.

### 3.5. Determination of the Protonation Constants

The details of the experiments using (traditional) potentiometric titration are presented in the Supporting Information. The results of the determination by Sirius instrument are shown in the Supporting Information. Potentiometric titrations of the compounds were performed with the PCA200 apparatus (Sirius Analytical Instruments Ltd, Forest Row, East Sussex, UK).

### 3.6. Elemental Analysis

Elemental analyses (C, H, N) were accomplished with a ThermoQuest CE Instruments EA 1110-CHNS-O elemental analyzer (CE Instruments, Milan, Italy). Phosphorus contents of compounds were determined at 880 nm by Jasco V-530 spectrophotometer using molybdenum blue method as described above. Solid samples were decomposed with nitric acid by the microwave digestion technique using CEM MDS-81D Microwave System prior to phosphorus determination. 

### 3.7. Thermal Analysis

Thermal decomposition paths with evaluation of crystal water content were obtained parallel with two instruments; Perkin Elmer STA 6000 thermogravimetric TG/DTA and Perkin Elmer TGA 7 analyzers (Perkin Elmer, Massachusetts, USA). Measurements were carried out in an open platinum crucible (the higher 5 mm crucible was used in TG/DTA and a shallower 2 mm crucible in TGA 7) under air atmosphere (flow rate of 45 mL/min) with a heating rate of 5 °C/min over the temperature range of 25−700 °C. The temperature calibration of STA 6000 was made using melting points of indium (156.60 °C) and zinc (419.5 °C) standards. For TGA 7 magnetic Curie-point calibration technique (Alumel, Ni, Perkalloy, Fe) was used. The weight balance of both instruments was calibrated by measuring a standard weight of 50 mg at room temperature. The sample weights used in the measurements were about 4–8 mg, and for each bisphosphonate, it was attempted to keep the same on both instruments. The melting/dehydration/decomposition behavior of the compounds was also visually monitored using a Stuart Scientific SMP3 melting point apparatus. 

## 4. Conclusions

In summary, ten aminobisphosphonates, H_2_N(CH_2_)_n_C(OH)[P(O)(OH)_2_]_2_**2**–**10** with variable chain lengths were prepared under similar conditions. As an exception compound **1** was isolated as a monosodium salt, whereas the other compounds were crystallized in their acidic forms. The common physicochemical and spectroscopic data of the bisphosphonates were measured. Only compounds **6** (n = 8) and **8** (n = 10) contained one molecule of water of crystallization. The solubility of the compounds was dependent on the chain length, since compounds **1**–**6** (n = 2–8) were soluble in gram per liter quantities, compound **7** in hundreds of milligrams per liter and the rest of the compounds only in tens of milligrams per liter. The decreasing trend in water solubility was most likely caused by the increasing lipophilicity of the compounds in parallel with the increase in alkyl chain length. As expected, temperature had also a clear effect on solubility, which was about doubled for compounds **2**, **4** and **5**, when the temperature was increased from 7.6 °C to 50.0 °C. However, pH had only a minimal effect on solubility of compounds **2**, **4** and **6**, while compounds **8** and **9** displayed minimal solubility at pH 2. The pK_a_ values were determined accurately for the most soluble compounds **1**–**5**, the pK_a1_-pK_a5_ values of which were in general 1, 2, 6, 10 and 12, respectively. 

The ^1^H-NMR spectra for the studied compounds were complicated due to the prochiral R-CH_2_-C(OH)P_2_ fragment. Detailed ^1^H-NMR spectral analyses were performed for compounds **1**–**5** indicating that only pamidronate exhibited any remarkable intramolecular hydrogen bonding. The other NMR chemical shifts and coupling constants in both liquid ^13^C, ^31^P-NMR and solid state ^13^C, ^15^N and ^31^P-NMR samples were typical for these types of compounds. Thermoanalytical studies of these compounds revealed their relatively low thermal stability as the majority of the compounds degraded at about 200 °C, most likely via a deamination process. 

## Supplementary Materials

Supplementary materials can be accessed at: http://www.mdpi.com/1420-3049/17/9/10928/s1.
