# Supplementary material for: Systematic Study of the Physicochemical Properties of a Homologous Series of Aminobisphosphonates
Source: Molecules. 2012 Sep 12;17(9):10928–45. doi: 10.3390/molecules170910928 (PMC6268373; doi:10.3390/molecules170910928)

Supporting Information

Systematic Study of Physicochemical Properties of Homologous Series of Aminobisphosphonates

Aino-Liisa Saari [1], Helena Hyvönen [2], Manu Lahtinen [3], Markku Ylisirniö [1], Petri Turhanen [1], Erkki Kolehmainen [3], Sirpa Peräniemi [1] and Jouko Vepsäläinen [1,*]

[1] School of Pharmacy, University of Eastern Finland, Biocenter Kuopio, P.O. Box 1627, FIN-70211 Kuopio, Finland

[2] Laboratory of Inorganic Chemistry, Department of Chemistry, University of Helsinki, P.O. Box 55, FIN-00014 Helsinki, Finland

[3] Department of Chemistry, University of Jyväskylä, P.O. Box 35, FIN-40014 Jyväskylä, Finland

Figure S1. The effect of length of the side chain on aqueous solubility at 21 °C.

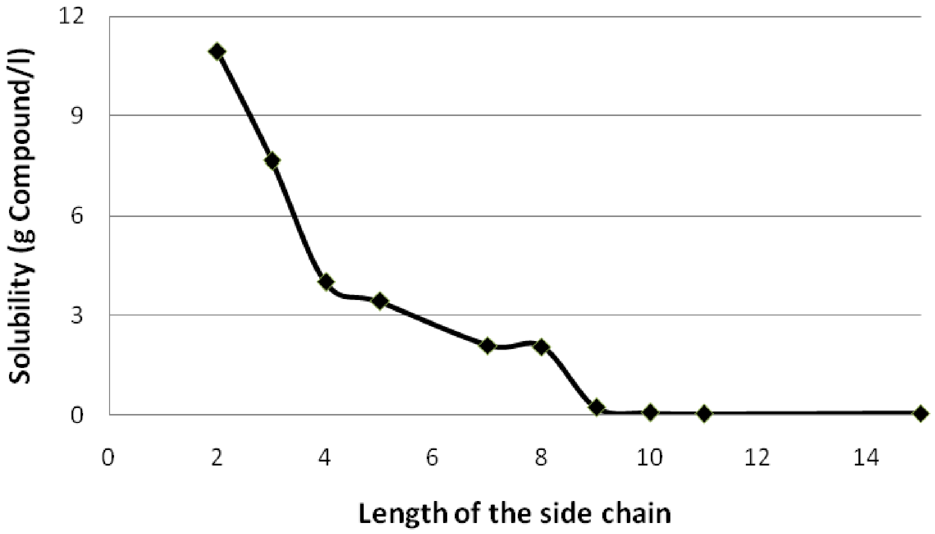

Table S1. The effect of temperature on aqueous solubility (mg/L) of aminoalkylbis(phosphonates) **2**, **4** and **5** (agitation time 24 h).

**Temperature**	**Solubility (g/L) of Compound**		
**(°C)**	**2**	**4**	**5**
4	6.36	4.47	1.85
7.6	8.41	4.60	1.90
21	9.55	5.17	2.10
30	10.76	5.77	2.27
40	12.86	7.62	2.40
50	15.06	9.55	3.45

Table S2. The effect of pH on aqueous solubility (mg/L) of aminoalkylbis(phosphonates) **2**, **4**, **6**, **8** and **9** (agitation time 30 min).

	**Solubility (g/L) of Compound**				
**pH**	**2**	**4**	**6**	**8**	**9**
0,5	9.93	6.34	2.10	0.18	0.13
1	7.61	3.84	1.15	0.08	0.08
2	7.66	2.90	0.86	0.05	0.07
3	7.53	2.97	1.17	0.08	0.08
4	7.51	3.09	1.08	0.18	0.14
5	7.44	3.22	1.11	0.23	0.20
6	7.32	3.39	1.23	0.27	0.21
7	8.39	3.42	1.19	0.29	0.20
8	8.00	3.53	1.23	0.25	0.24
9	7.92	3.08	1.19	0.29	0.25
10	7.92	3.18	1.20	0.40	0.40
11	7.53	3.38	1.27	0.37	0.47

Determination of the Protonation Constants Using (traditional) Potentiometric Titration. Reagents: Aqueous 0.1 M NaOH was prepared from Titrisol ampoules (Merck) and 0.1 M HNO_3 from Convol ampoules (BDH). The background solution was prepared by dissolving $NaNO_3$ in deionised water. The $NaNO_3$ and NaCl used in reference electrode were p.a. grade (Merck). The water used in the dilutions and titration solutions was purified with Milli-RO and Milli-Q water purification systems (Millipore).

Potentiometric measurements: The protonation equilibria was studied in aqueous 0.1 M $NaNO_3$ at 25.0 ± 0.1 °C through a series of potentiometric EMF titrations carried out with a Schott-Geräte GmbH titrator TPC2000 and utilizing titration software TR600 version 5.02. The cell arrangement for the measurement of the hydrogen ion concentration [H+] was as follows:

$$-\text{RE} \mid \text{equilibrium solution} \mid \text{GE}+ \quad (1)$$

where GE denotes a glass electrode (Schott N2680), and RE is Hg, Hg_2Cl_2 || 0.01 M NaCl, 0.09 M $NaNO_3$. Expression (2) is valid assuming that the activity coefficients are constant.

$$E = E_0 + 59.157 \log[H^+] + j_H [H^+] + j_{OH} [OH^-] \quad (2)$$

The cell parameter E_0 and the liquid junction coefficient j_H, valid in acidic solutions, were determined for each titration by adding a known amount of HNO_3 to the background electrolyte. The value of the liquid junction coefficient j_{OH}, valid in basic solutions, was determined periodically. Only stable EMF readings were used in the calculations.

During the measurements of the protonation equilibria, aqueous 0.1 M NaOH or 0.1 M HNO_3 was added to the solution. The initial concentrations of ligands varied within the limits 0.7 mM ≤ C_L ≤ 4.5 mM. Four to eight independent titrations were carried out for each ligand. The number of data points used in the calculation of the stability constants varied from 305 to 551 in the pH ranges 1.87–11.99.

Data treatment: Protonation and deprotonation of the ligands were controlled by addition of HNO_3 or NaOH. The curves of Z_H versus pH were drawn to visualize the experimental data sets. Z_H describes the average number of H^+ ions added or liberated per mole of ligand and is given by the relationship

$$Z_H = (C_H - [H^+] + k_w[H^+]^{-1})/C_L \quad (3)$$

where C_H denotes the total concentration of protons calculated over the zero level H_2L^{2-}.

In the evaluation of the equilibrium constants, the following two-component equilibria were utilized:

$$H_2L^{2-} \rightleftharpoons H_{2-x}L^{(2+x)-} + xH^+,\ x = 1–2;\ \beta_{-x} \quad (4)$$

$$xH^+ + H_2L^{2-} \rightleftharpoons H_{x+2}L^{x-2},\ x = 1–3;\ \beta_x \quad (5)$$

Mathematical analysis of the systems involves a search for protonation models and equilibrium constants that best describe the experimental data. The calculations were carried out with the computer program SUPERQUAD (Gans, P.; Sabatini, A.; Vacca, A. *J. Chem. Soc. Dalton Trans.* **1985**, 1195-1200). The sample standard deviation Σ and the χ^2 statistics used as criteria in the selection of the models were those provided by the program. As a means to improve the confidence level, the error limits for log β values determined in this study are reported as three times the standard deviation estimated by the program.

Figure S2. Z_H *vs.* pH for protonation of compounds (a) **1**; (b) **2**; (c) **3**; (d) **4** and (e) **5**.

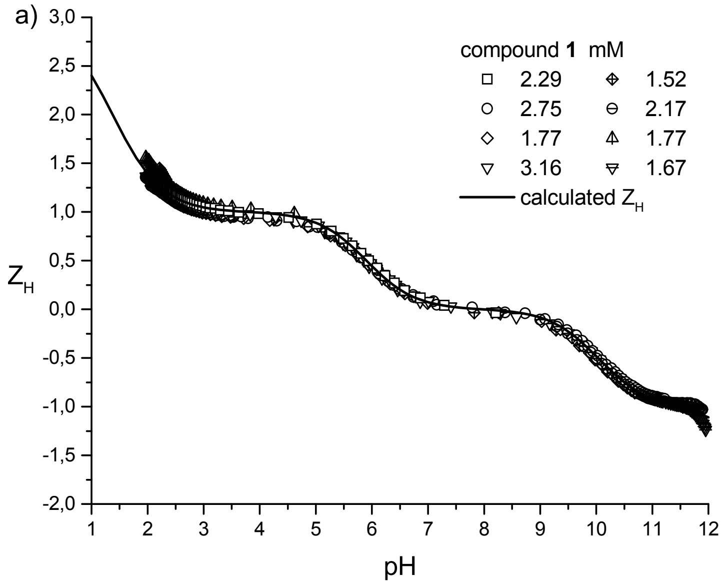

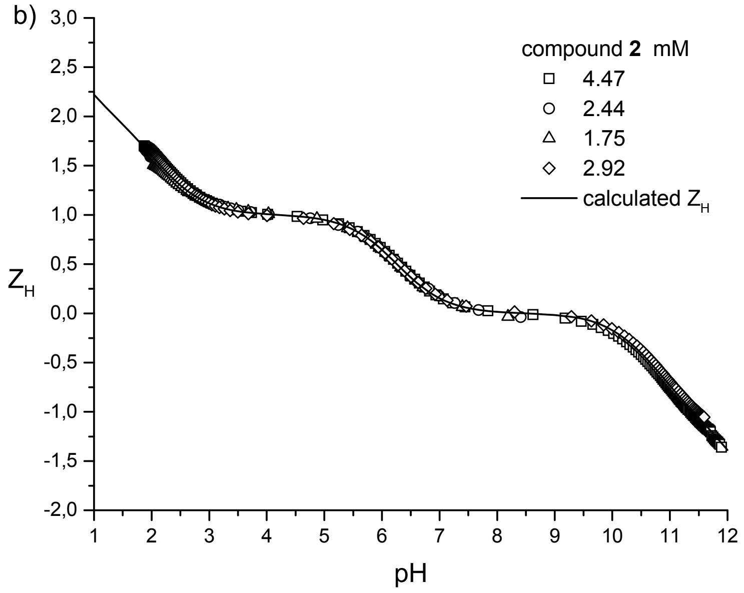

Figure S2. *Cont.*

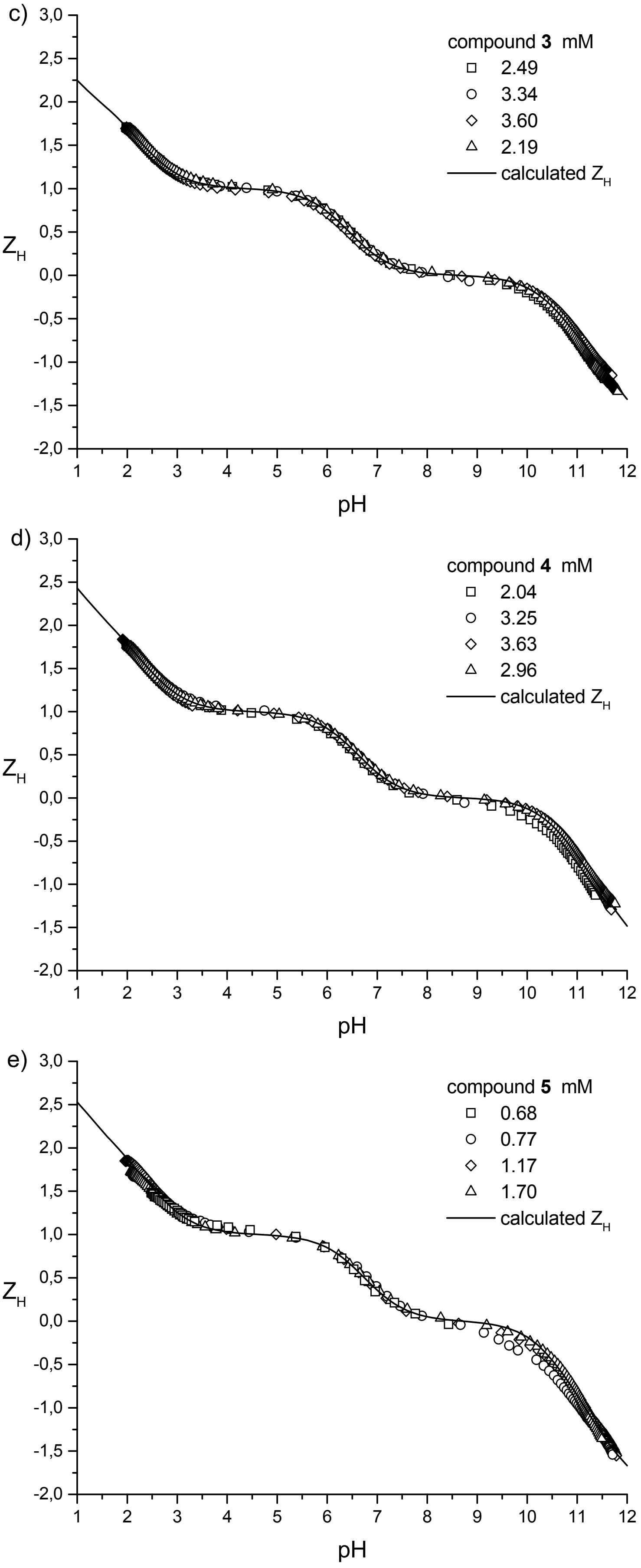

Figure S3. Percentage distribution of the protonation stages of compounds (a) **1**; (b) **2**; (c) **3**; (d) **4** and (e) **5** *versus* pH.

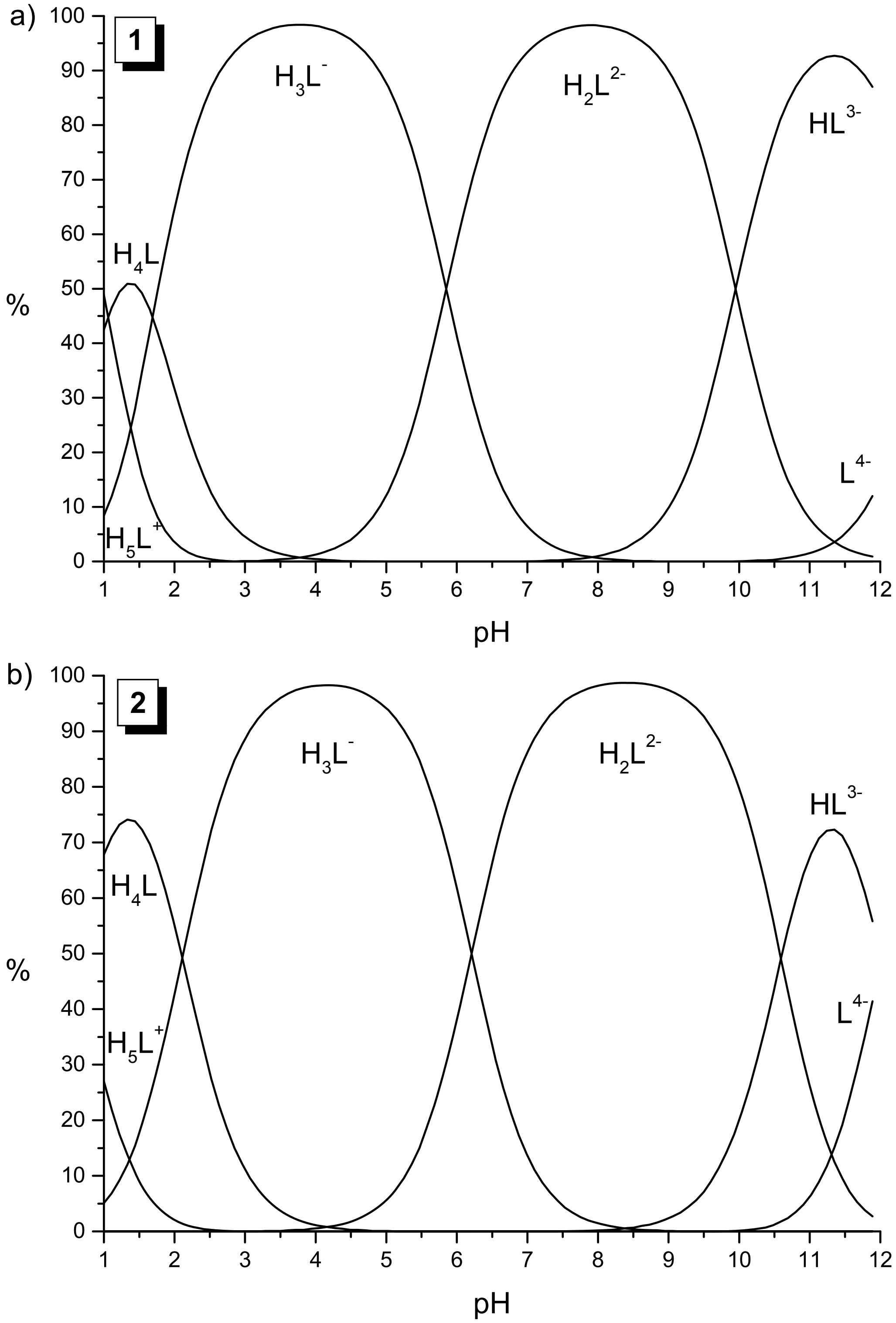

Figure S3. *Cont.*

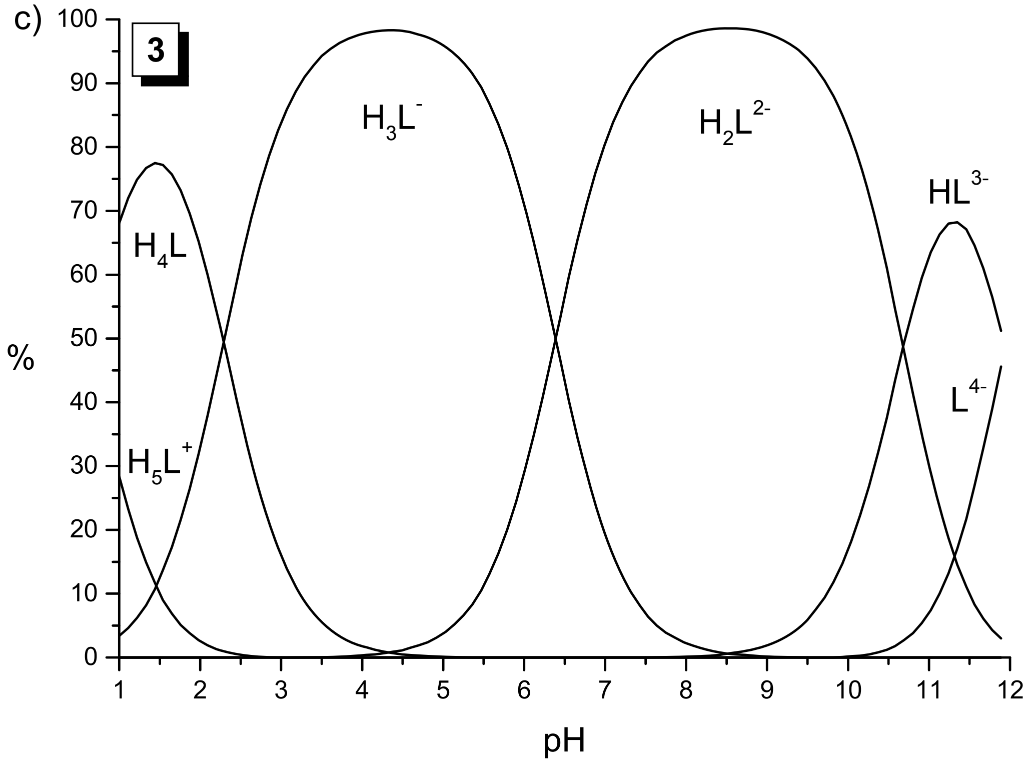

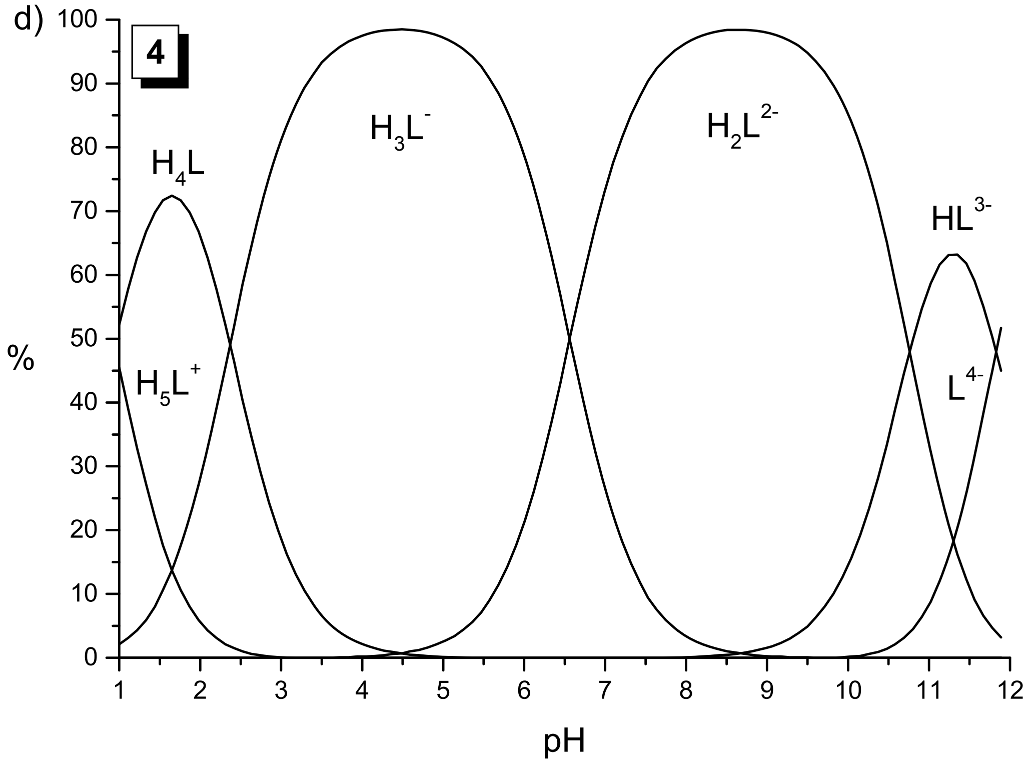

Figure S3. *Cont.*

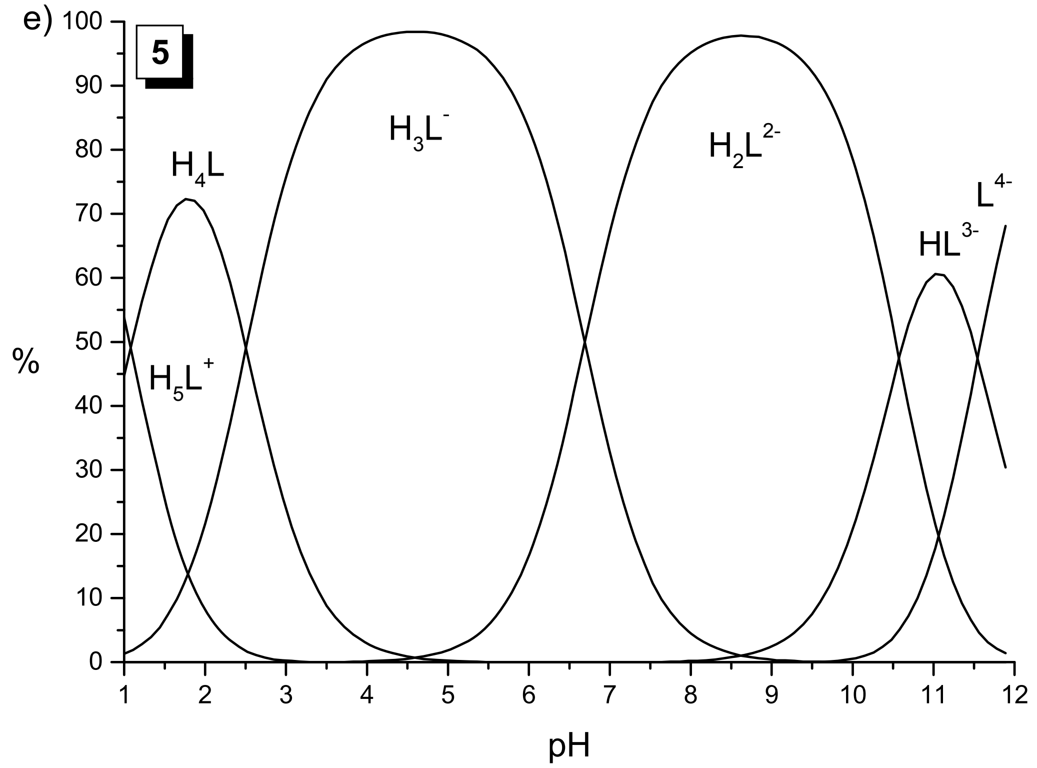

Table S3. The overall protonation of compounds **1**, **2**, **3**, **4**, and **5** in 0.1 M $NaNO_3$ aqueous solution at 25 °C (equations 4 and 5, zero level H_2L^{2-}).

x (eqn 4, 5)	Compound 1 *logβ$_x$ ± 3σ*	Compound 2 *logβ$_x$ ± 3σ*	Compound 3 *logβ$_x$ ± 3σ*	Compound 4 *logβ$_x$ ± 3σ*	Compound 5 *logβ$_x$ ± 3σ*	Formula
−2	−22.90 ± 0.23	−22.82 ± 0.02	−22.83 ± 0.03	−22.80 ± 0.02	−22.32 ± 0.02	L^{4-}
−1	−10.04 ± 0.03	−10.69 ± 0.01	−10.78 ± 0.02	−10.86 ± 0.02	−10.67 ± 0.03	HL^{3-}
1	5.90 ± 0.02	6.26 ± 0.01	6.44 ± 0.02	6.62 ± 0.02	6.75 ± 0.03	H_3L^-
2	7.60 ± 0.05	8.38 ± 0.02	8.74 ± 0.03	9.00 ± 0.03	9.27 ± 0.05	H_4L
3	8.66 ± 0.30	8.98 ± 0.20	9.36 ± 0.51	9.94 ± 0.12	10.35 ± 0.24	H_5L^+
χ^2 / Σ	19.15 / 1.95	15.99 / 1.21	17.14 / 1.85	35.57 / 1.72	15.81 / 1.10	
points / titrations	551 / 8	378 / 4	385 / 4	331 / 4	305 / 4	

Table S4. The comparison of pK_a values of **1**–**5** measured by Sirius instrument and traditional potentiometric titration (TPT).

**Compound**	**1**		**2**		**3**		**4**		**5**	
**pKa**	**Sirius**	**TPT**	**Sirius**	**TPT**	**Sirius**	**TPT**	**Sirius**	**TPT**	**Sirius**	**TPT**
1		1.06		0.60		0.62		0.94		1.08
2	2.32	1.70	2.18	2.12	1.92	2.30	2.99	2.38	3.10	2.52
3	5.93	5.90	6.17	6.26	2.42	6.44	6.36	6.62	6.46	6.75
4	10.05	10.04	10.70	10.69	6.40	10.78	6.82	10.86	7.12	10.67
5	12.54	12.86	12.21	12.13	15.68 *	12.05	13.19 *	11.94	10.77	11.65

* mystic value.

Details for Solid State NMR Measurements. The contact times for the CPMAS experiments were 2 ms for ^{13}C, 3 ms for ^{15}N and 5 ms for ^{31}P. The relaxation delay was 5 s and the spin rate was 10 KHz. In the high-power broad band ^{1}H decoupling the pulse program was spinal64 for carbon-13 and nitrogen-15 and tppm15 for phosphorus-31. The number of scans was typically hundred for carbon-13, thousands for nitrogen-15 and less than one hundred for phosphorus-31. The ^{13}C and ^{15}N chemical shifts were referenced those of glycine (176.03 ppm for carbon-13 and -345.25 ppm for nitrogen-15) measured prior to each sample. The ^{31}P-NMR chemical shifts are referenced that of sodium alendronate measured before.

Table S5. Liquid state ^{13}C as well as solid state (SS) ^{15}N and ^{31}P CPMAS NMR chemical shifts.

	**C-1** [1]	**C-2** [2]	**C-3** [3]	**C-4**	**C-5**	**C-6**	**C-7**	**C-8**	**C-9**	**C-10**	^{31}**P**	^{15}**N**
**1**	78.7 (133.8 Hz)	40.0	40.9	-	-	-	-	-	-	-	18.46	-
SS [4]	72.3 (145.8 Hz)	32.5	37.6								9.07, 7.19	−345.1
**2**	79.3 (134.5 Hz)	36.2	30.2	44.6	-	-	-	-	-	-	18.89	-
SS [4]	73.85 (147.5)	30.3	23.0	41.3							14.32, 4.74	−345.7
**3**	79.2 (133.5 Hz)	38.1	24.1	34.3	42.9	-	-	-	-	-	18.92	-
SS [4]	73.7 ()	29.3	20.5	26.9	41.2						15.59, 2.04	−345.5
**4**	79.6 (134.4 Hz)	39.0	26.9	30.4	34.7	43.5	-	-	-	-	19.12	-
SS [4]	74.6 (147.9 Hz)	33.7	20.8	24.1	26.8	37.8					14.16, 0.55	−344.5
**5**	78.9 (132.4 Hz)	38.4	27.0	33.0	31.5	29.0	33.6	43.2	-	-	19.05	-
SS [4]	73.2 (Hz)	36.4	24.7	33.1	29.8	27.4	33.1	39.7			7.36, 5.51	−347.9
**6**	79.6 (134.6 Hz)	39.0	27.2	33.2	31.7	31.9	28.9	34.7	43.4	-	19.30	-
SS [4]	72.5 [5]	[5]	[5]	[5]	[5]	[5]	[5]	[5]	40.7		6.07, 2.94	−342.1
**7**	79.7 (134.5 Hz)	39.1	27.2	33.3	31.9	31.9	31.5	28.9	34.7	43.4	19.25	-
SS [4]	73.2 (147.9 Hz)	36.5	24.8	[5]	[5]	[5]	[5]	[5]	[5]	39.8	7.51, 5.27	−347.9
**8**	79.6 (134.4 Hz)	39.1	27.2	33.3	31.9	31.9	31.6	31.4	28.	34.6	19.33	-
SS [4]	[5]	[5]	[5]	[5]	[5]	[5]	[5]	[5]	[5]	[5] [6]	[7]	−342.0 [8]
**9**	79.6 (134.4 Hz)	39.1	27.2	33.3	31.9	31.9	31.6	31.5	31.4	28.9	19.35	-
SS [4]	73.1 (149.6 Hz)	36.5	24.8	[5]	[5]	[5]	[5]	[5]	[5][9]	39.8	5.47, 5.19 [10]	−348.1
**10** [4]	73.0 (149)	36.2	[5]	[5]	[5]	[5]	[5]	[5]	[5]	[5]	7.41, 5.23 [10]	−348.0 [11]

1) $^{1}J_{CP}$ coupling in parenthesis; 2) in liquid the state, the signal broadened due to $^{2}J_{CH}$ coupling but the value was less than 1 Hz; 3) $^{3}J_{CH}$ between 5.0-5.7 Hz; 4) chemical shift in solid state measurements; 5) not assigned due to broad and overlapping signals; 6) NCH_2 shift at 43.4 ppm; 7) 10.5-2.4 mixture of several forms; 8) Minor signal at 346.1 ppm; 9) NCH_2-CH_2 shifts at 43.4 and 34.6 ppm; 10) containing also some minor forms; 11) Minor signal at 346.1 ppm.

Experimental for Supportive X-ray Powder Diffraction Measurements. The X-ray powder diffraction data were measured with PANalytical X'Pert PRO diffractometer in Bragg–Brentano geometry using step–scan technique and Johansson monochromator to produce pure Cu $K_{\alpha 1}$ radiation (1.5406 Å; 45 kV, 30 mA). Lightly hand-ground powder sample was prepared on a silicon-made zero–background holder using petrolatum jelly as an adhesive. The data was collected from a spinning sample by X'Celerator detector in 2θ range of 3–70° with a step size of 0.017° and counting times of 240 s per step. Programmable divergence slit (PDS) was used in automatic mode to set irradiated length on sample to 10 mm together with fixed 15 mm incident beam mask. Soller slits of 0.02° rad were used on incident and diffracted beam sides together with anti–scatter slits of 4° and 8.7 mm, respectively.

The diffraction data were converted from automatic slit mode (ADS) to the fixed slit mode (FDS) data in PANalytical HighScore Plus v. 2.2d software package before further analyses. The ICDD PDF-2 powder diffraction database (International Centre for Diffraction Data, ICDD-PDF2, Release 2007, Newton Square, PA, USA) implemented in Highscore Plus was used for the search-match phase identification analyses.

Figure S4. X-ray powder diffraction pattern of TG residue (at 700 °C) of compound **1**. Characteristic peak positions of $H_2Na_2P_2O_7$ are indicated by red vertical drop lines.

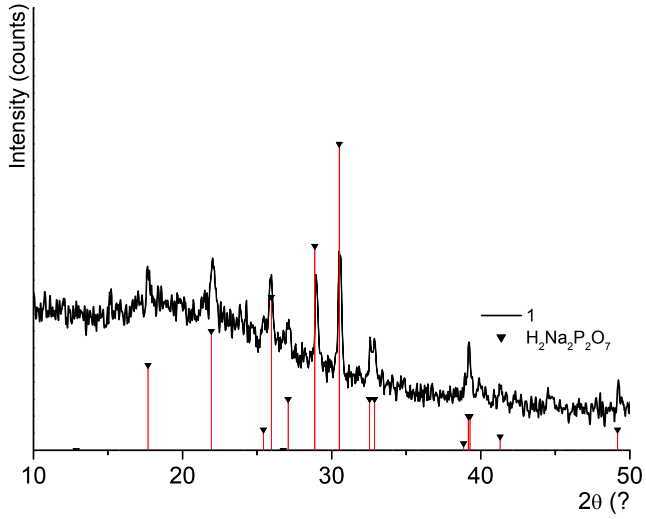

Table S6. Thermal properties of compounds **1–10**.

Comp.	MW	Exp. Δwt-% (%)	Calculated Δwt-% (%)	Temp. range of 1st cleavage (°C)	T_d [b] (°C)	Residual Weights (%)
**1**	257.05	**7.09**	6.55 (1 NH_3)	134–199 (149)	149	66.9
**1** **	257.05	**7.50**	6.55 (1 NH_3)	102–203 (155)	155	61.6
**2**	249.1	**7.02**	6.84 (1 NH_3)	168–252 (222)	222	22.1
**2** **	249.1	**7.02**	6.84 (1 NH_3)	166–253 (225)	225	1.8
**3**	263.12	**6.85**	6.47 (1 NH_3)	198–252 (217)	217	22.9
**3****	263.12	**6.75**	6.47 (1 NH_3)	195–265 (224)	224	1.6
**4**	277.15	**5.11**	6.14 (1 NH_3)	196–260 (208)	208	13.8
**4** **	277.15	**5.03**	6.14 (1 NH_3)	183–251 (215)	215	1.2
**5**	305.2	**4.10**	5.58 (1 NH_3)	182–212 (193)	193	9.7
**5** **	305.2	**4.07**	5.58 (1 NH_3)	175–222 (197)	197	1.4
**6**	337.23	**4.89**	5.34 (1 H_2O)	33–175 (77)	190	28.2
**6** **	337.23	**5.10**	5.34 (1 H_2O)	33–173 (84)	191	20.7
**7**	333.26	**6.18**	5.11 (1 NH_3)	175–211 (185)	185	16.7
**7** **	333.26	**5.83**	5.11 (1 NH_3)	161–227 (189)	189	0

Table S6. *Cont.*

Comp.	MW	Exp. Δwt-% (%)	Calculated Δwt-% (%)	Temp. range of 1st cleavage (°C)	T_d [b] (°C)	Residual Weights (%)
**8**	365.3	**4.22**	4.93 ($1H_2O$)	50–146 (73)	199	10.0
**8** **	365.3	**4.65**	4.93 ($1H_2O$)	39–149 (70)	204	0.8
**9**	361.31	**5.25**	4.71 ($1 NH_3$)	163–232 (185)	185	10.3
**9** **	361.31	**4.96**	4.71 ($1 NH_3$)	155–221 (191)	191	0
**10**	417.42	**5.10**	4.08 ($1 NH_3$)	150–248 (178)	178	13.5
**10** **	417.42	**4.11**	4.08 ($1 NH_3$)	146–245 (181)	181	0.3

** = measured with a shallow crucible by TGA 7; [a] lower limit of the *T* range taken at 0.5 Δwt-%; [b] thermal decomposition and melting transition coexist on all compounds.

Figure S5. TG-curves of compounds **1–10** measured by a heating rate of 5 °C/min under air atmosphere. The measurements made with a taller pan are indicated by black solid lines and with a shallow pan by red dashed lines, respectively. The DTA signals are indicated by solid blue lines.

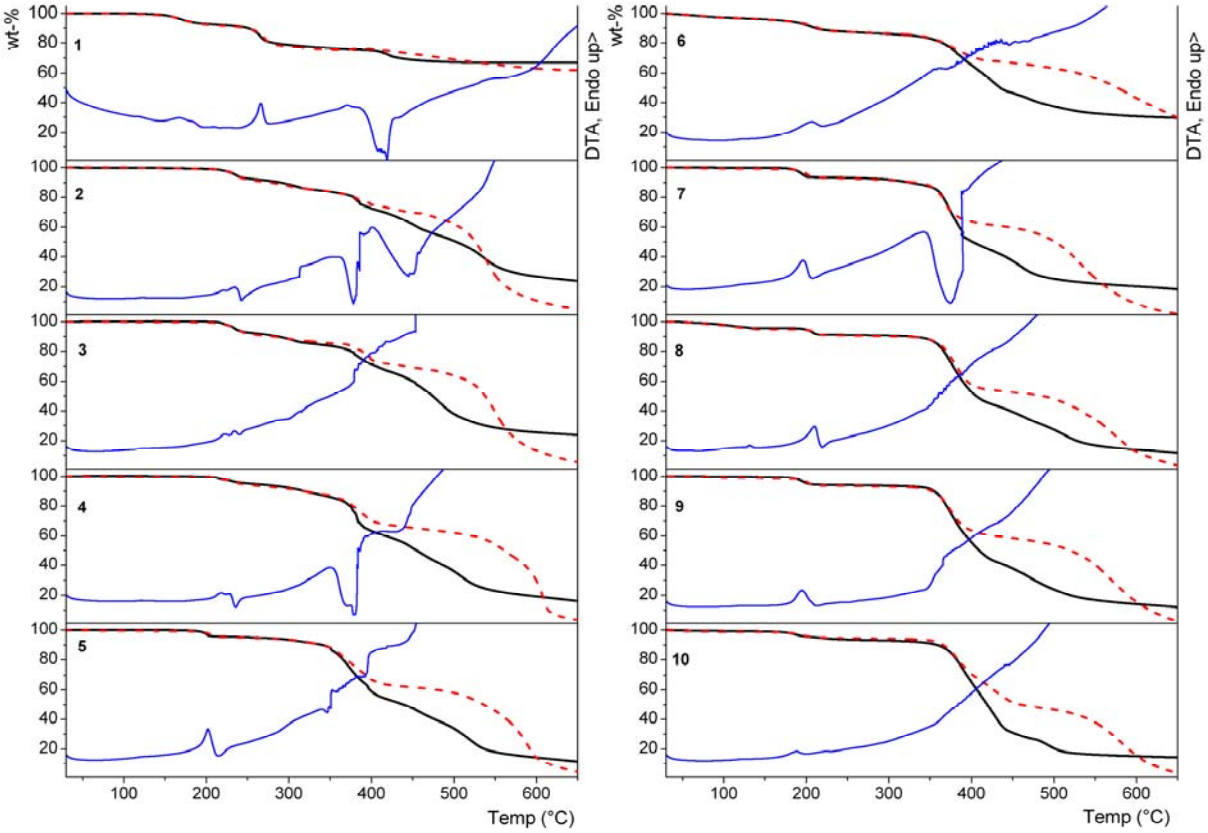

Supplement: Supplementary file 1 [file molecules-17-10928-s001.pdf]
